# Supplementary material for: Adolescent stress remodels synapses in the sensory thalamus and impairs tactile discrimination in mice
Source: Commun Biol. 2025 Nov 25;8:1678. doi: 10.1038/s42003-025-09075-8 (PMC12647837; doi:10.1038/s42003-025-09075-8)
Supplement: Supplementary file 1 — Supplementary Information [file 42003_2025_9075_MOESM1_ESM.pdf]

Supplementary Information

Adolescent stress remodels synapses in the sensory thalamus and impairs tactile discrimination in mice.

Hisako Nakayama and Mariko Miyata

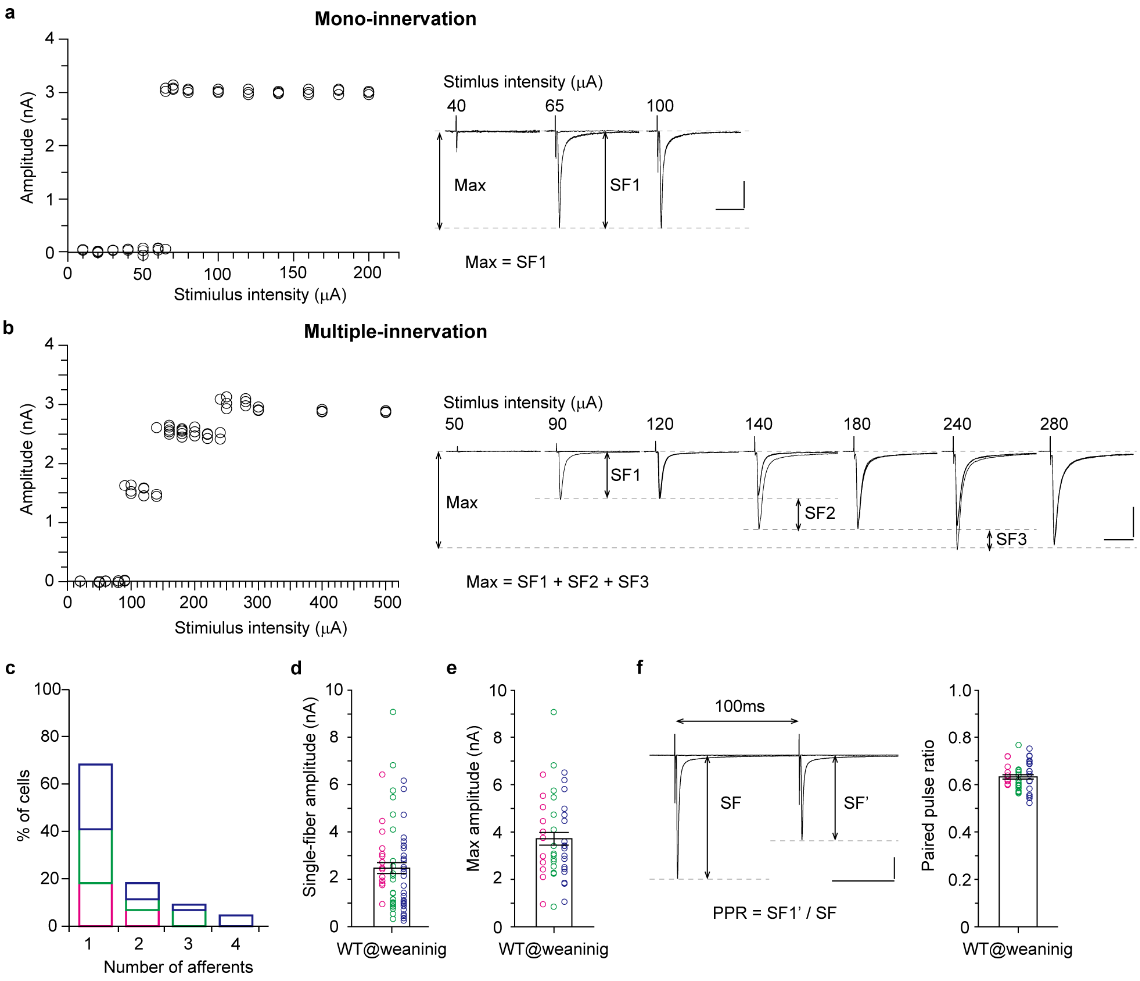

Supplementary Fig. 1: The mono-innervation at MLF-VPM synapses has been completed by the weaning day of P21.

a-b, Relationship between EPSC amplitude and intensity of electrical stimulation to the MLF observed in VPM neurons determined to be mono-innervation (**a**) and multiple-innervation (**b**) (left) and EPSCs recorded at several stimulus intensities in the left plots. Three to five trials were performed at each stimulus intensity. EPSC waveforms are superimposed over 3-5 trials. The number of steps in EPSCs observed during stimulus intensity increase was used as an afferent number. Max is the maximum amplitude of EPSCs recorded in each cell; SF1-3 are the amplitudes of the incremental EPSC stepwise changes. Scale bars: 5ms and 1 nA. **c,** Distribution histograms of afferent numbers (n = 44 cells). **d,** Plots of SF-amplitude (n = 65 fibers) and Max-amplitude (n = 43 cells). **e,** Plots of paired-pulse ratio (PPR, n = 65 fibers). Data were obtained from 3 wild-type male mice aged P20-P22. Boxes represent mean values with error bars of ± SEM (**d-f**). Data from different mice were represented by different colors (**c-f**).

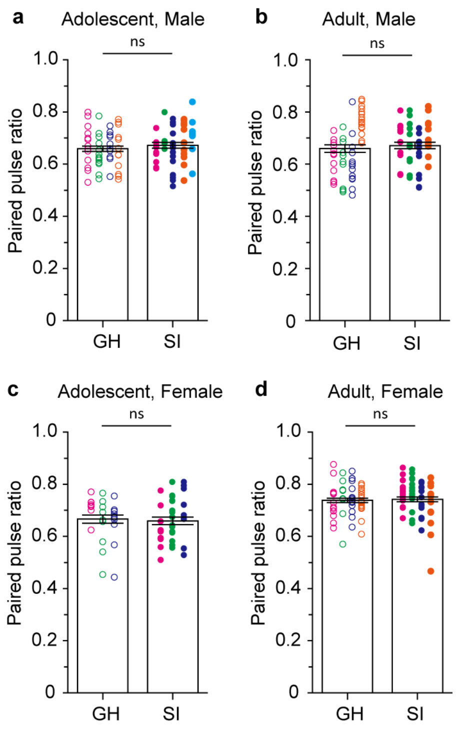

Supplementary Fig. 2: The PPR remains unchanged by SI rearing during adolescence or adulthood in both sexes.

a, b, Plots of PPRs obtained from male mice kept in GH or SI conditions during adolescence (**a**, GH, n = 49, N =4; SI, n = 49, N = 5, p = 0.305 by U-test) and adulthood (**b**, GH, n = 52, N =4; SI, n = 45, N = 4, p = 0.548 by U test). **c, d,** Plots of PPRs obtained from female mice in GH or SI conditions during adolescence (**c**, GH, n = 28, N =3; SI, n = 35, N = 3, p = 0.422 by U-test) and adulthood (**d**, GH, n = 55, N =4; SI, n = 57, N = 4, p = 0.417 by U-test). Boxes represent mean values with error bars of ± SEM. Different colors represented data from other mice. Data were obtained from the same mice/cells used for Fig. 1 and Extended Data Fig. 4.

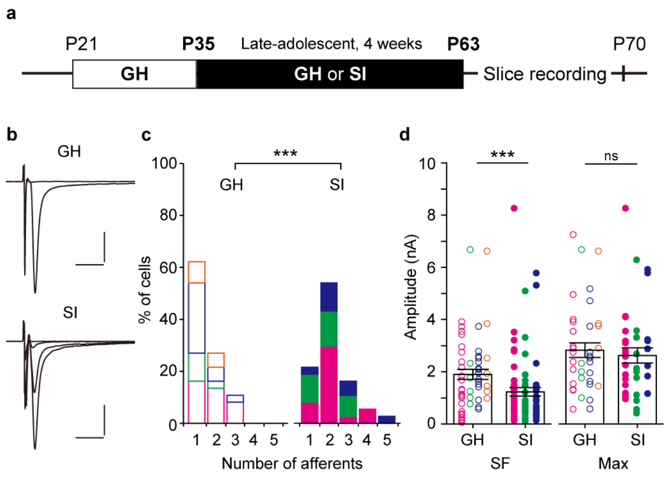

Supplementary Fig. 3: SI in late adolescence remodels MLF-VPM synapses.

a, Male mice were weaned at P21 and kept in a GH condition for one week and then divided into GH or SI conditions at P35, and reared for 4 weeks in each condition (GH, N = 4; SI, N = 3). **b,** MLF-VPM EPSCs. Scale bars: 5 ms and 1 nA. Vh = -70 mV. **c,** Histograms of afferent numbers (GH, n = 37; SI, n = 37, $***p < 0.001$ by *U*-test). **d,** Plots of SF- and Max-amplitude (SF-amplitude: GH, n = 55; SI, n = 79, $***p < 0.001$ by *U* test; Max-amplitude: GH, n = 37; SI, n = 37, $p = 0.527$ by *U*-test). Each point represents the amplitude of each fiber and each VPM neuron, respectively. Boxes represent mean values with error bars of ± SEM. Data from different mice were represented with different symbols.

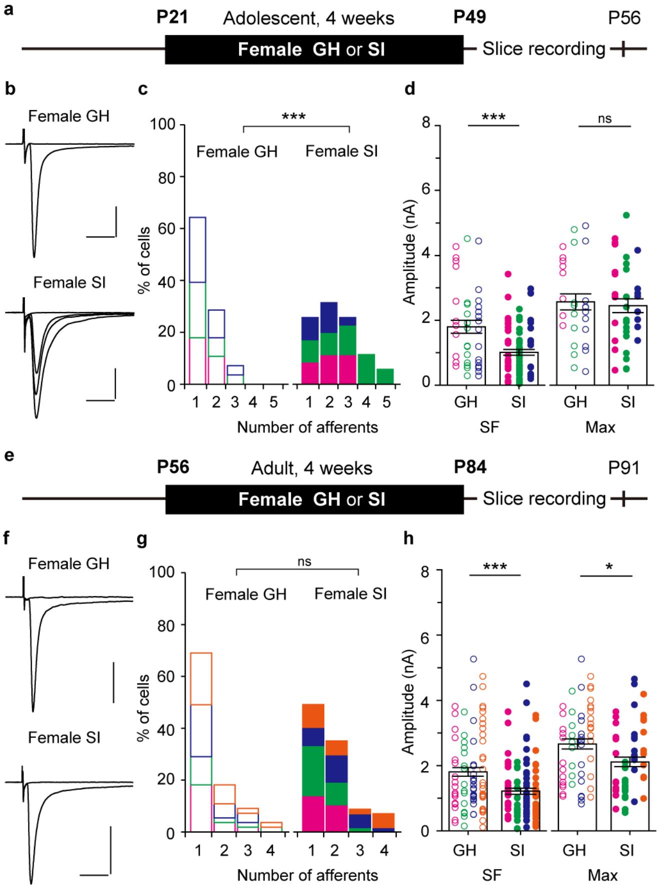

Supplementary Fig. 4: Adolescent SI but adult SI rearing remodels MLF-VPM synapses in female mice.

a, Female mice were reared in GH or SI conditions for 4 weeks from P21 (Female GH, N = 3; Female SI, N = 3). **b,** MLF-VPM EPSCs. **c,** Histograms of afferent numbers (Female GH, n = 28; Female SI, n = 35, ***$p < 0.001$ by *U*-test). **d,** Plots of SF- and Max-amplitude (SF-amplitude: Female GH, n = 40; Female SI, n = 85, ***$p < 0.001$ by *U*-test; Max-amplitude: Female GH, n = 28; Female SI, n = 35, $p = 0.740$ by *U*-test). **e,** All female mice were kept in GH from P21 to P56 and divided into GH or SI conditions for 4 weeks (Female GH, N = 4; Female SI, N = 4). **f,** MLF-VPM EPSCs. **g,** Histograms of afferent numbers (Female GH, n = 55; Female SI, n = 57, $p = 0.053$ by *U*-test). **h,** Plots of SF- and Max-amplitude (SF-amplitude: Female GH, n = 81; Female SI, n = 99, ***$p < 0.001$ by *U*-test; Max-amplitude: Female GH, n = 55; Female SI, n = 57, *$p = 0.010$ by *U*-test). Each point represents the amplitude of each fiber and each VPM neuron, respectively. Boxes represent mean values with error bars of ± SEM. Data from different mice were represented with different symbols. Scale bars: 5 ms and 1 nA (**b, f**). Vh = -70 mV.

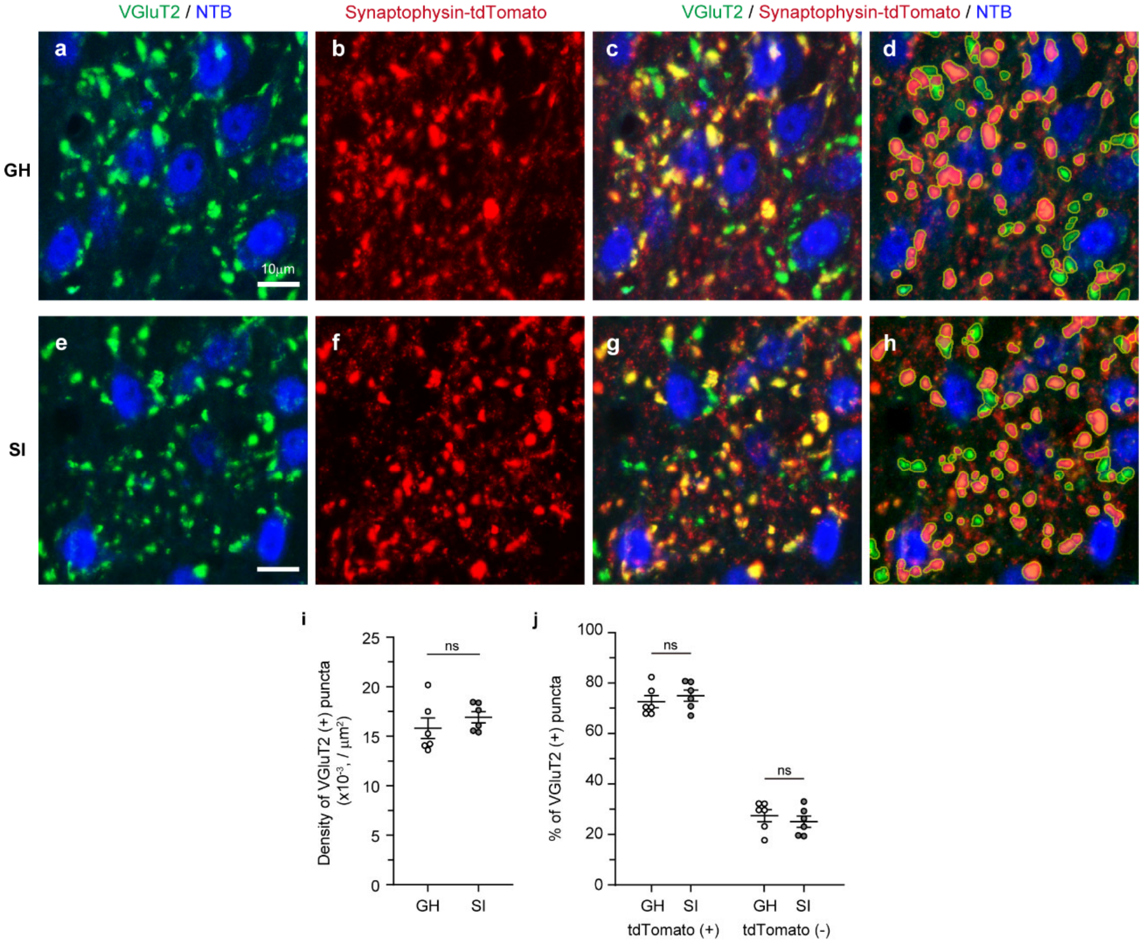

Supplementary Fig. 5: Adolescent SI does not increase synapses originating from non-whisker regions in the brain stem.

a-h, Fluorescent images of VGluT2 (green), synaptophysin-tdTomato (red), and NeuroTrace 435/455 (blue, Nissl stain) in the VPM of a GH (**a-d**) and a SI (**e-h**) mouse. Images are z-stacks of 16 slices taken every 0.3 μm. Scale bars: 10 μm. **d, h,** An example of analysis results. Using image analysis software (BZ-H4A and BZ-H4C, KEYENCE), VGluT2-positive puncta were extracted and outlined with yellow lines, and tdTomato-positive puncta among them are colored pink. The number of VGluT2-positive puncta outlined by yellow lines and the number of pink-colored ones were counted, and the density and overlap ratio were calculated and plotted on **i** and **j**. **i,** Density of VGluT2-positive puncta (GH, N = 6; SI, N = 6, df = 10, t = -0.935, p = 0.372 by Student's t-test). **j,** Ratios of tdTomato-positive and -negative puncta among VGluT2-positive puncta (tdTomato(+): GH, N = 6; SI, N = 6, df = 10, t = -0.720, p = 0.488 by Student's t-test; tdTomato(-): GH, N = 6; SI, N = 6, df = 10, t = 0.720, p = 0.488 by Student's t-test).

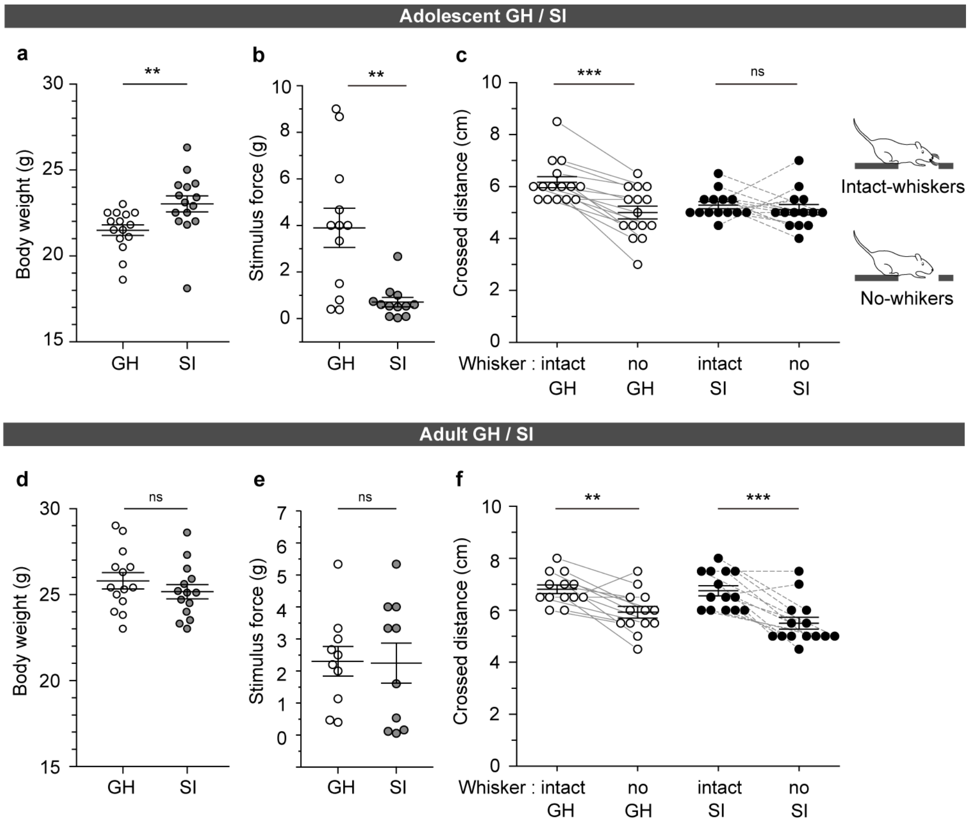

Supplementary Fig. 6: Adolescent, but not adult, SI hypersensitizes tactile perception of whiskers and impairs whisker-using behavior.

Mice were weaned at P21 (**a-c**) or 8 weeks old (**d-f**) and reared in GH or SI conditions for 4 weeks before the behavioral tests. **a, d**, Body weight of mice at the behavioral tests (Adolescent: GH, N = 15; SI, N = 14, $^{**}p = 0.003$ by U-test; Adult: GH, N = 13; SI, N = 14, $p = 0.397$ by U-test). **b, e**, Threshold stimulus intensities at which mice responded to mechanical stimulation of von Frey filament (Adolescent: both GH and SI, N = 12, $^{**}p = 0.004$ by U-test; Adult: both GH and SI, N = 10, $^{**}p = 0.094$ by U-test). **c, f,** Maximum gap distance that mice with intact or no whiskers could successfully cross (Adolescent: intact whiskers, N = 15, df = 14, t = 6.718, $^{***}p < 0.001$ by paired t-test; no whiskers, N = 14, df = 13, t = 0.673, $p = 0.513$ by paired t-test; Adult: intact whiskers, N = 13, df = 12, t = 3.888, $^{**}p = 0.002$ by paired t-test; no whiskers, N = 14, df = 13, t = 5.235, $^{***}p < 0.001$ by paired t-test). Each point represents data from a single mouse. Gray lines connect data from the same mouse.

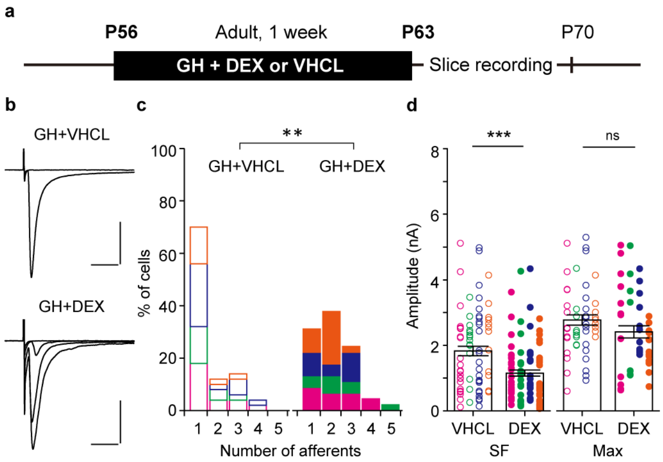

Supplementary Fig. 7: Adult MLF-VPM synapses are remodeled in response to pharmacological activation of GRs.

a, Mice were kept in GH condition from P21. DEX (5 mg/kg body weight) or VHCL was administered intraperitoneally once a day from P56 to P63 (DEX, N = 4; VHCL, N = 4). **b,** Representative MLF-VPM EPSC. Scale bars: 5 ms and 1 nA. Vh = -70 mV. **c,** Histograms of afferent numbers (GH+VHCL, n = 50; GH+DEX, n = 45, $**p = 0.001$ by *U*-test). **d,** Plots of SF- and Max-amplitude (SF-amplitude: GH+VHCL, n = 76; GH+DEX, n = 94, $***p < 0.001$ by *U*-test; Max-amplitude: GH+VHCL, n = 50; GH+DEX, n = 45, $p = 0.084$ by *U*-test). Scale bars: 5 ms and 1 nA. Each point represents the amplitude of each fiber and each VPM neuron, respectively. Boxes represent mean values with error bars of ± SEM. Data from different mice were represented with different symbols.

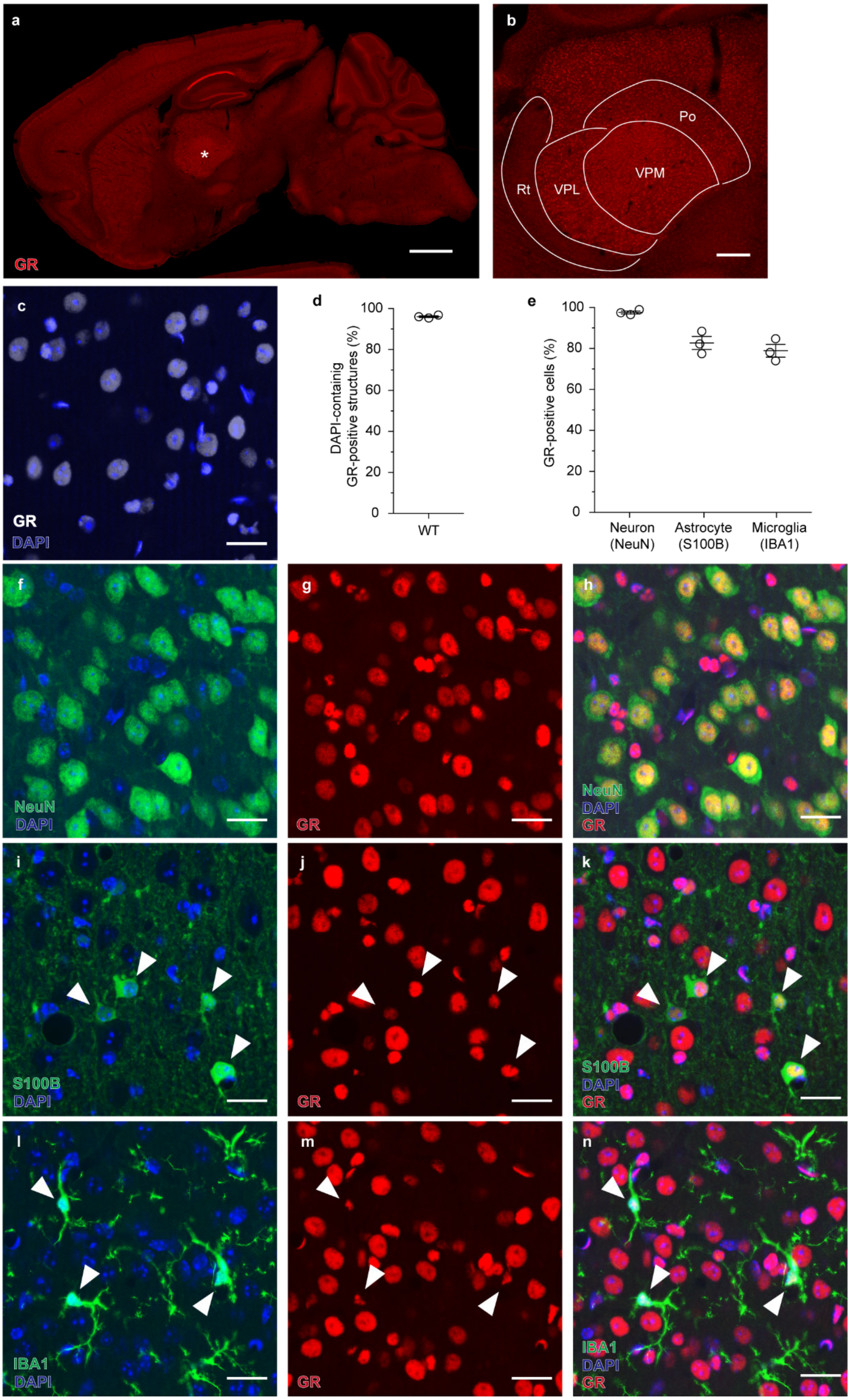

a
GR
b
Po
Rt
VPL
VPM
c
GR
DAPI
d
DAPI-containig GR-positive structures (%)
100
80
60
40
20
0
WT
e
GR-positive cells (%)
100
80
60
40
20
0
Neuron (NeuN)
Astrocyte (S100B)
Microglia (IBA1)
f
NeuN
DAPI
g
GR
h
NeuN
DAPI
GR
i
S100B
DAPI
j
GR
k
S100B
DAPI
GR
l
IBA1
DAPI
m
GR
n
IBA1
DAPI
GR

Supplementary Fig. 8: GRs are expressed in most neurons, astrocytes, and microglia in the VPM.

a, Immunohistochemical staining for GRs in a male wild-type mouse at P21. The asterisk indicates the region of the VPM. **b,** Enlarged view of the VPM and its surroundings. The boundaries of the neuronal nuclei (white lines) are added based on the mouse brain atlas [1]. VPM: Ventral posteromedial nucleus of the thalamus; VPL: Ventral posterolateral nucleus of the thalamus; PO: Posterior complex of the thalamus; Rt: Reticular nucleus of the thalamus. **c,** Overlapping images of GR (gray) and DAPI (blue). **d,** Percentage of GR-positive structures that contain DAPI among all GR-positive structures (N = 3). **e,** Percentage of GR-positive cells in neurons, astrocytes, and microglia, identified by expressing NeuN, S100B, and IBA1, respectively (N = 3 for each cell type). **f, i, l,** Overlaid images of DAPI (blue) and NeuN (green, **f**), S100B (green, **i**), and IBA1 (green, **l**). **g, j, m,** Images for GR. **h, k, n,** Superimposed images of DAPI (blue) and cell-type markers (green; NeuN in **h**, S100B in **k**, and IBA1 in **n**) along with GR (red). White arrowheads indicate GR-positive astrocytes and microglia (**i**-**n**). In the representative images in **f**-**h**, all neurons were GR-positive. Scale bars: 1 mm (**a**), 250 µm (**b**), and 20 µm (**c**, **f**-**n**).

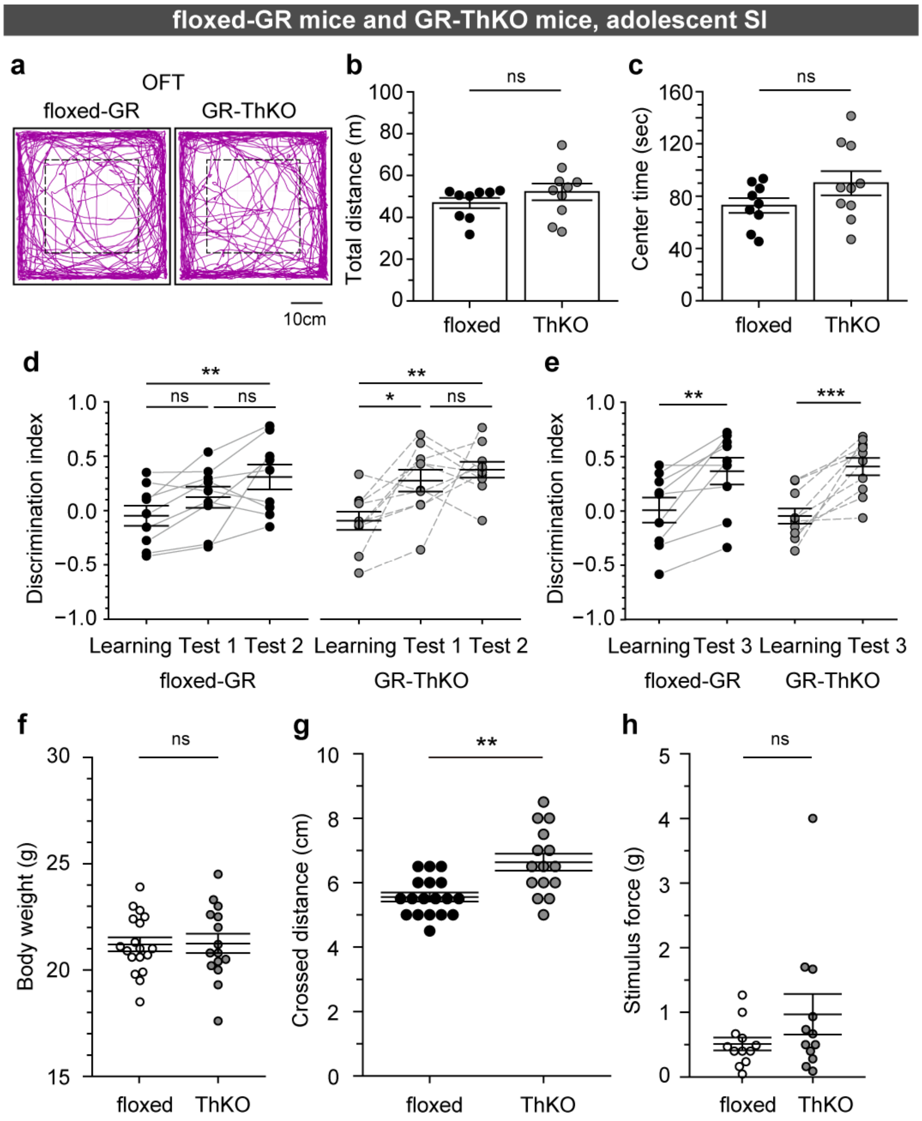

Supplementary Fig. 9: Abnormalities in beard-related behaviors caused by adolescent SI were partially suppressed in GR-ThKO mice.

Male floxed-GR and GR-ThKO mice were weaned at P21 and reared in GH or SI conditions for 4 weeks before the behavioral tests. **a**, Mouse trajectories during OFT. **b**, Total distance traveled during OFT (floxed-GR, N = 9; GR-ThKO, N = 10, df = 17, t = -1.103, $p = 0.285$ by Student's t-test). **c**, Time spent in the center area during OFT (floxed-GR, N = 9; GR-ThKO, N = 10, df = 17, t = -1.535, p = 0.143 by Student's t-test). **d**, Discrimination index in the tNORT (floxed-GR: N = 9; Between sessions: $F(2, 16) = 6.519$, $p < 0.008$ by one-way RM ANOVA; Learning vs. Test 1: $p = 0.102$; Learning vs. Test 2: $*p = 0.007$; Test 1 vs. Test 2: $p = 0.151$ by Holm-Sidak post hoc test; GR-ThKO: N = 10; Between sessions: $F(2, 18) = 7.075$, $p = 0.005$ by

one-way RM ANOVA; Learning vs. Test 1: *$p = 0.023$; Learning vs. Test 2: **$p = 0.007$; Test 1 vs. Test 2: $p = 0.459$ by Holm-Sidak post hoc test). **e**, Discrimination index in the NORT (floxed-GR: N = 9, df = 8, t = -3.934, **$p = 0.004$ by paired *t*-test; GR-ThKO: N = 10, df = 9, t = -5.048, ***$p < 0.001$ by paired *t*-test). Data from the same mouse are connected by a gray line (floxed-GR) or a dashed line (GR-ThKO). **f**, Body weight of mice at the behavioral tests (floxed-GR, N = 18; GR-ThKO, N = 15, $p = 1.000$ by *U*-test). **g**, Maximum gap distance with intact whiskers (floxed-GR, N = 18; GR-ThKO, N = 15, **$p = 0.002$ by *U*-test). **h**, Threshold stimulus intensities in von Frey test (both floxed-GR and GR-ThKO, N = 12, $p = 0.259$ by *U*-test). Vertical lines indicate mean ± SEM.

Supplementary reference

1 Paxinos, K. B. J. F. G. *The Mouse Brain in Stereotaxic Coordinates Third Edition*. (Elsevier Inc., 2007).
