# Supplementary material for: Adolescent stress remodels synapses in the sensory thalamus and impairs tactile discrimination in mice
Source: Commun Biol. 2025 Nov 25;8:1678. doi: 10.1038/s42003-025-09075-8 (PMC12647837; doi:10.1038/s42003-025-09075-8)
Supplement: Supplementary file 2 — Description of Additional Supplementary Files [file 42003_2025_9075_MOESM2_ESM.pdf]

Description of Additional Supplementary files

File name: Supplementary Data 1

Description: The source data behind the graphs in the paper
