# Supplementary material for: Adolescent stress remodels synapses in the sensory thalamus and impairs tactile discrimination in mice
Source: Commun Biol. 2025 Nov 25;8:1678. doi: 10.1038/s42003-025-09075-8 (PMC12647837; doi:10.1038/s42003-025-09075-8)
Supplement: Supplementary file 4 — Reporting Summary [file 42003_2025_9075_MOESM4_ESM.pdf]

nature portfolio

Corresponding author(s): Hisako Nakayama
Mariko Miyata
COMMSBIO-25-0316B

Last updated by author(s): Sep 30, 2025

Reporting Summary

Nature Portfolio wishes to improve the reproducibility of the work that we publish. This form provides structure for consistency and transparency in reporting. For further information on Nature Portfolio policies, see our Editorial Policies and the Editorial Policy Checklist.

Statistics

For all statistical analyses, confirm that the following items are present in the figure legend, table legend, main text, or Methods section.

n/a	Confirmed	
☐	☒	The exact sample size (*n*) for each experimental group/condition, given as a discrete number and unit of measurement
☐	☒	A statement on whether measurements were taken from distinct samples or whether the same sample was measured repeatedly
☐	☒	The statistical test(s) used AND whether they are one- or two-sided<br>*Only common tests should be described solely by name; describe more complex techniques in the Methods section.*
☐	☒	A description of all covariates tested
☐	☒	A description of any assumptions or corrections, such as tests of normality and adjustment for multiple comparisons
☐	☒	A full description of the statistical parameters including central tendency (e.g. means) or other basic estimates (e.g. regression coefficient) AND variation (e.g. standard deviation) or associated estimates of uncertainty (e.g. confidence intervals)
☐	☒	For null hypothesis testing, the test statistic (e.g. *F*, *t*, *r*) with confidence intervals, effect sizes, degrees of freedom and *P* value noted<br>*Give P values as exact values whenever suitable.*
☒	☐	For Bayesian analysis, information on the choice of priors and Markov chain Monte Carlo settings
☒	☐	For hierarchical and complex designs, identification of the appropriate level for tests and full reporting of outcomes
☒	☐	Estimates of effect sizes (e.g. Cohen's *d*, Pearson's *r*), indicating how they were calculated

Our web collection on statistics for biologists contains articles on many of the points above.

Software and code

Policy information about availability of computer code

Data collection	Immunohistochemical images were acquired using BZ-H4XI (Image site meter module), Z-H4XD (Multistack module), BZ-H4XF (Sectioning module) for KEYENCE BZ-X800. Electrophysiology data were acquired using Patchmaster (HEKA Elektronik). Mice movements were captured with FlyCapture2 camera software 2.9.3.11 (Photonics).
Data analysis	Fit Master (HEKA Elektronic), Sigma Plot 14.5 (SYSTAT), Excel (Mirosoft), Origin Pro (Origin Lab), BZ-H4C and BZ-H4A (Hybrid cell count software, KEYENCE), ANY-maze (Stoelting)

For manuscripts utilizing custom algorithms or software that are central to the research but not yet described in published literature, software must be made available to editors and reviewers. We strongly encourage code deposition in a community repository (e.g. GitHub). See the Nature Portfolio guidelines for submitting code & software for further information.

Data

Policy information about availability of data

All manuscripts must include a data availability statement. This statement should provide the following information, where applicable:

- Accession codes, unique identifiers, or web links for publicly available datasets
- A description of any restrictions on data availability
- For clinical datasets or third party data, please ensure that the statement adheres to our policy

The datasets in the current study will be available as Supplementary Data before publication.

Research involving human participants, their data, or biological material

Policy information about studies with human participants or human data. See also policy information about sex, gender (identity/presentation), and sexual orientation and race, ethnicity and racism.

Reporting on sex and gender	N/A
Reporting on race, ethnicity, or other socially relevant groupings	N/A
Population characteristics	N/A
Recruitment	N/A
Ethics oversight	N/A

Note that full information on the approval of the study protocol must also be provided in the manuscript.

Field-specific reporting

Please select the one below that is the best fit for your research. If you are not sure, read the appropriate sections before making your selection.

☒ Life sciences ☐ Behavioural & social sciences ☐ Ecological, evolutionary & environmental sciences

For a reference copy of the document with all sections, see nature.com/documents/nr-reporting-summary-flat.pdf

Life sciences study design

All studies must disclose on these points even when the disclosure is negative.

Sample size	The sample size was estimated based on previous reports. We conducted the experiments using more than 3 mice per group.
Data exclusions	Electrophysiological experiments were not analyzed if a noisy recording condition was present.
Replication	The electrophysiological data from the group-housed wild-type mice (GH) are reproducible across experiments (Fig. 1, 4, Extended Data Fig. 1, 2, and 3).<br><br>The frequency of mono innervation and the EPSC amplitude recorded from VPM neurons in the GH mice (Fig. 1, 4, Extended Data Fig. 1, 2, and 3) are consistent with the results of previous studies:<br>Takeuchi et al. Large-Scale Somatotopic Refinement via Functional Synapse Elimination in the Sensory Thalamus of Developing Mice. J Neurosci. 34 (4):1258-1270 (2014)<br>Arsenault and Zhang. Developmental remodelling of the lemniscal synapse in the ventral basal thalamus of the mouse. J Physiol. 573:121–132 (2006)<br><br>The density of VGluT2-positive puncta and ratios of tdTomato-positive and -negative puncta among VGluT2 puncta in the GH mice (Extenden Data Fig. 4) are consistent with the results of a previous study:<br>Takeuchi et al. Large-Scale Somatotopic Refinement via Functional Synapse Elimination in the Sensory Thalamus of Developing Mice. J Neurosci. 34 (4):1258-1270 (2014)
Randomization	To randomize the rearing conditions, experiments were conducted using littermate mice and data were allocated into experimental groups.
Blinding	The electrophysiological experiments using transgenic mice were performed blindly to their genotype. Behavioral experiments were conducted by laboratory technicians blind to experimental conditions and mouse genotypes.

Reporting for specific materials, systems and methods

We require information from authors about some types of materials, experimental systems and methods used in many studies. Here, indicate whether each material, system or method listed is relevant to your study. If you are not sure if a list item applies to your research, read the appropriate section before selecting a response.

Materials & experimental systems

n/a	Involved in the study
☐	☒ Antibodies
☒	☐ Eukaryotic cell lines
☒	☐ Palaeontology and archaeology
☐	☒ Animals and other organisms
☒	☐ Clinical data
☒	☐ Dual use research of concern
☒	☐ Plants

Methods

n/a	Involved in the study
☒	☐ ChIP-seq
☒	☐ Flow cytometry
☒	☐ MRI-based neuroimaging

Antibodies

Antibodies used

VGluT2 pAb (GP) (VGluT2-GP-Af810, RRID, AB_2341096), Frontier Institute (now NIttobo).
Anti-Glucocorticoid Receptor antibody [EPR19621] (ab183127), Abcam
Anti-S100beta antibody (287 006), Synaptic Systems.
Anti-Iba1 goat (011-27991), FUJIFILM Wako Pure Chemical Corporation.
Anti-NeuN Antibody, clone A60 (MAB377), Merk.
These five primary antibodies described above were used in immunohistochemistry.

Validation

All antibodies used in the study are validated in the manufacturer's website in NIttobo (https://nittobo-nmd.co.jp/pdf/reagents/VGluT2.pdf) and abcam (https://www.abcam.co.jp/products/primary-antibodies/glucocorticoid-receptor-antibody-epr19621-ab183127.html), Synaptic Systems (https://www.sysy.com/product/287006), FUJIFILM Wako Pure Chemical Corporation (https://labchem-wako.fujifilm.com/jp/product/detail/W01W0101-2799.html), and Merk (https://www.merckmillipore.com/JP/ja/product/Anti-NeuN-Antibody-clone-A60,MM_NF-MAB377).

Animals and other research organisms

Policy information about studies involving animals; ARRIVE guidelines recommended for reporting animal research, and Sex and Gender in Research

Laboratory animals

C57BL/6NCrSlc mice (Japan SLC, Inc, RRID: MGI:5295404), Krox20-Cre mice (Egr2tm2(cre)Pch/J, RRID:IMSR_JAX:025744), Ai34D mice (B6;129S-Gt(ROSA)26Sortm34.1(CAG-Syp/tdTomato)Hze/J, RRID:IMSR_JAX:012570), floxed-GR mice (C57BL/6-Nr3c1<tm1.1Himo>, #RBRC10693), and 5HTT-cre mice (C57BL/6J-Tg(Slc6a4-cre)208Ito, #RBRC10598) aged P21-P91 were used.

Wild animals

No wild animals were used in this study.

Reporting on sex

We used male mice in all experiments but female mice in Extendended data fig. 2.

Field-collected samples

No field-collected samples were used in this study.

Ethics oversight

All experiments were conducted according to the guidelines of the experimental animal ethics committees and the biosafety committee for living modified organisms of Tokyo Women's Medical University, the guiding principles for the care and use of experimental animals of the physiological society in Japan and the Japan Neuroscience Society.

Note that full information on the approval of the study protocol must also be provided in the manuscript.

Plants

Seed stocks

N/A

Novel plant genotypes

N/A

Authentication

N/A
